# No major tumorigenic role for *β*-catenin in serrated as opposed to conventional colorectal adenomas

**DOI:** 10.1038/sj.bjc.6601070

**Published:** 2003-07-01

**Authors:** T Yamamoto, K Konishi, T Yamochi, R Makino, K Kaneko, T Shimamura, H Ota, K Mitamura

**Affiliations:** 1 Second Department of Internal Medicine, Showa University School of Medicine, Tokyo, Japan; 2 Second Department of Pathology, Showa University School of Medicine, Tokyo, Japan; 3 Clinical Laboratory, Showa University School of Medicine, Tokyo, Japan; 4 Department of Microbiology and Immunology, Showa University School of Medicine, Tokyo, Japan

**Keywords:** serrated adenoma, *β*-catenin, colorectal adenoma, serrated polyp, Wnt signal transduction

## Abstract

Intracellular redistribution of *β*-catenin through mutation of the adenomatous polyposis coli (*APC*) gene has been proposed as an early tumorigenic event in most colorectal tumours. In serrated adenoma (SA), a newly recognised subtype of colorectal adenoma, *APC* mutations are uncommon, and the contribution of *β*-catenin to tumorigenesis remains unclear. We compared intracellular localisation of *β*-catenin and presence of mutations in exon 3 of *β*-catenin between 45 SAs, with 71 conventional adenomas (CADs), and eight carcinomas invading the submucosa (SCAs). Widespread or focal nuclear *β*-catenin expression was demonstrated in 7% of SAs (three out of 45), 61% of CADs (43 out of 71), and 88% of SCAs (seven out of eight). Cytoplasmic immunostaining for *β*-catenin was demonstrated in 16% of SAs (seven out of 45), 77% of CADs (55 out of 71), and 88% of SCAs (seven out of eight). No mutation in exon 3 of *β*-catenin was found in SAs or SCAs, while 7% of CADs (five out of 71) had *β*-catenin mutations. No nuclear or cytoplasmic expression of *β*-catenin was observed in the hyperplastic or conventionally adenomatous epithelium of mixed-type SAs. These findings suggest that *β*-catenin mutation is unlikely to contribute to the tumorigenesis in SA, and that intracellular localisation of *β*-catenin may not be associated with an early event of the tumour progression in most SAs.

The adenoma–carcinoma sequence of colorectal carcinogenesis requires an accumulation of genetic alterations. Inactivation of the adenomatous polyposis coli (*APC*) suppressor gene is an initial event in the development of most colorectal tumours (Kinzler and Vogelstein, 1996). This inactivation leads to a decrease in APC-dependent degradation of the oncoprotein *β*-catenin, thereby increasing its cytoplasmic abundance (Korinek *et al*, 1997; Morin *et al*, 1997). The *β*-catenin protein is involved in two functions: cell-to-cell adhesion and mediation of the Wingless/Wnt signal transduction pathway (Behrens *et al*, 1996; Barth *et al*, 1997); the latter action is associated with the oncogenic potential of this protein. This pathway involves stabilisation of the cytoplasmic pool of *β*-catenin, translocation of *β*-catenin to the nucleus, complex formation between *β*-catenin and transcription factors of the T-cell factor (Tcf) family, and activation of this transcriptional complex resulting in constitutive transcriptional activation of downstream target genes that regulate cell proliferation or apoptosis (Korinek *et al*, 1997; Morin *et al*, 1997). Recently, c-*myc* (He *et al*, 1998) and cyclin D1 (Tetsu and McCormick, 1999) have been identified as target genes of the *β*-catenin/Tcf complex. Such signal transduction involving *β*-catenin plays a critical role in colorectal carcinogenesis.

Recently, mutation in exon 3 of *β*-catenin has been identified in approximately half of colorectal tumours that lack *APC* mutations (Morin *et al*, 1997). These *β*-catenin mutations involve alterations of exon 3 of serine/threonine sites that normally are phosphorylated by glycogen synthatase kinese 3*β* (GSK-3*β*). As phosphorylation of these sites is apparently necessary for APC-induced degradation of *β*-catenin (Morin *et al*, 1997), the *β*-catenin mutations cause intracellular *β*-catenin accumulation, increasing formation of the transcription-activating *β*-catenin/Tcf complex.

Some authors recently have reported that hyperplastic colorectal polyps may in fact be neoplastic lesions because they include some of the genetic alterations seen in colorectal cancers (CRC), such as Ki-*ras* mutations (Otori *et al*, 1997) and microsatellite instability (Jass *et al*, 2000b). Serrated adenoma (SA), which histologically shows hyperplastic architecture and epithelial dysplasia, has been recognised as a new subtype of colorectal adenoma (Longacre and Fenoglio-Preiser, 1990). Given an incidence of high-grade dysplasia in SAs of 10–14.8% (Longacre and Fenoglio-Preiser, 1990; Matsumoto *et al*, 1999), SAs have been considered potential precursors of colorectal carcinoma. Some reports have concluded that colorectal carcinoma may be associated with SA (Hiyama *et al*, 1998; Makinen *et al*, 2001). On the other hand, SAs often occur in contiguity with typical hyperplastic polyps (Iino *et al*, 1999). While some investigators have suggested that genetic alterations in SAs differ from those in conventional adenomas (CADs) (Ajioka *et al*, 1998; Hiyama *et al*, 1998; Uchida *et al*, 1998; Iino *et al*, 1999; Jass *et al*, 2000a; Rashid *et al*, 2000; Dehari, 2001; Hawkins and Ward, 2001), the differences have not been specified.

Recently, Dehari (2001) and Uchida *et al* (1998) have reported that *APC* gene mutation is uncommon in SA. However, the contribution of *β*-catenin to tumorigenesis in SA remains unclear. The aim of this study was to examine intracellular localisation of *β*-catenin and to identify any mutations in exon 3 of *β*-catenin in SAs, by comparing the findings with those in CADs and carcinomas invading the submucosa (SCAs).

## MATERIALS AND METHODS

### Subjects

A total of 45 SAs from 43 patients who underwent endoscopic (*n*=44) or transanal resection (*n*=1) at Showa University Hospital between September 1997 and December 2001 were analysed in this study. CADs (*n*=71) and SCAs (*n*=8) obtained endoscopically or surgically were studied for comparison. Informed consent was obtained from all patients. We excluded patients who had familial adenomatous polyposis, hereditary nonpolyposis colorectal cancer (HNPCC), or hyperplastic polyposis (Jass and Burt, 2000).

### Histologic evaluation

**Figure 1:**
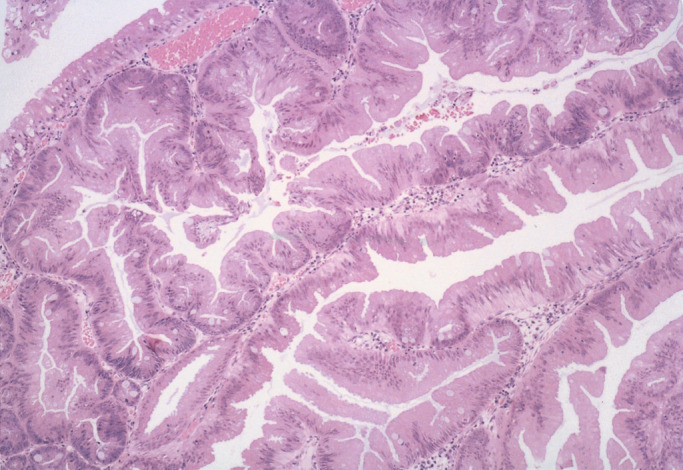
Histologic appearance of a typical SA showing serrated architecture and epithelial dysplasia. H&E staining.

Specimens resected or obtained by endoscopy were fixed in 10% buffered formalin for 24 h, dehydrated, and embedded in paraffin. Serial sections (3 *μ*m) were cut from the paraffin block to be prepared for haematoxylin and eosin (H&E) staining and immunostaining. All H&E-stained sections were reviewed by a senior pathologist (TY), who was blinded to colonoscopic findings. The histologic diagnosis of SA was based on the definition of Longacre and Fenoglio-Preiser (1990) (Figure 1

). Histologic findings of (1) a serrated architecture simulating a hyperplastic polyp, (2) goblet cell immaturity, (3) mitotic figures in the upper crypt zone, (4) prominence of nuclei, and (5) cytoplasmic eosinophilia were the criteria for inclusion. SAs were subclassified as pure or mixed depending on whether or not a component other than SA was present. Epithelial dysplasia without serrated architecture was classified as CAD. When the tumour cells had spread through the muscularis mucosae into the submucosa, the lesion was defined as carcinoma. Clinicopathologic characteristics of patients with SAs, CADs, and SCAs are shown in Table 1

**Table 1:** Clinicopathologic characteristics of patients with SAs, CADs, and SCAs

	**SAs**	**CADs**	**SCAs**
	**(*n*=45)**	**(*n*=71)**	**(*n*=8)**
Gender			
M/F	26/17	35/17	5/3
			
Age	60.0	64.2	60.0
(range)	(30–88)	(31–83)	(51–71)
			
Location			
Left-c	35	51	5
Right-c	10	20	3
			
Size (mm)			
<10	18	39	1
⩾10	27	32	7
			
Macroscopic type			
Polypoid	36	51	6
Superficial	9	20	2
			
Histologic dysplasia			
LGD	37	51	NA
HGD	8	20	
			
Histology of SA			
SA	24	NA	NA
SA+HYP	13		
SA+CAD	7		
SA+SCA	1		

SAs=serrated adenomas; CADs=conventional adenomas; SCAs=carcinomas invading the submucosa; HYP=hyperplastic polyps; M/F=male/female; Left-c=rectum, sigmoid colon, and descending colon; Right-c=transeverse colon, ascending colon, and caecum; LGD=low-grade dysplasia; HGD=high-grade dysplasia; NA=not applicable.

. No significant difference was noted in the distribution for gender, age, location, size, or macroscopic type between SA and CAD groups.

### Immunohistochemical detection of *β*-catenin

Deparaffinised, rehydrated sections were heated in a microwave oven in 0.01 mol l^−1^ sodium citrate buffer (pH 6.0) for 15 min to retrieve antigens. Endogenous peroxidase activity was inhibited by incubation with 0.3% hydrogen peroxidase in methanol for 5 min. Sections were incubated with anti-*β*-catenin antibody (1 : 300, Transduction Labs, Lexington, KY, USA) for 90 min. Sections were then incubated with horseradish peroxidase (HRP)-binding amino-acid polymer for 30 min (Histofine Simplestain MAX-PO kit, Nichrei, Tokyo, Japan). Colour was developed by staining with diaminobenzidine solution. Sections were lightly counterstained with haematoxylin. Phosphate-buffered saline (pH 7.2) was used for rinsing between each step.

### Evaluation of *β*-catenin immunostaining

**Figure 2:**
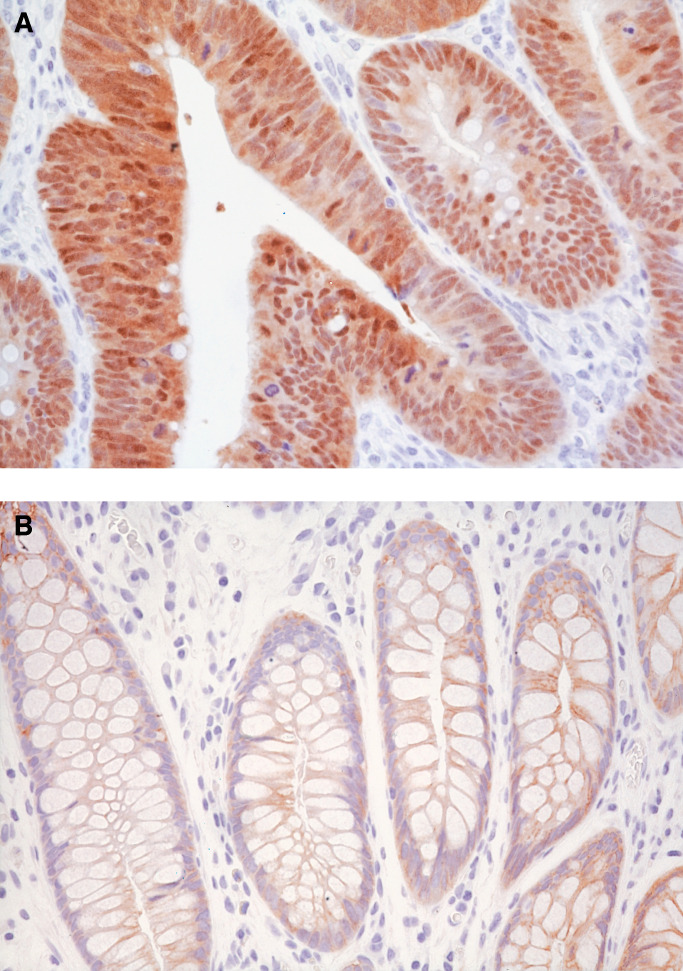
Immunohistochemical staining of *β*-catenin. (**A**) Widespread nuclear and cytoplasmic expression in a colonic adenoma with high-grade dysplasia. (**B**) Membrane expression of *β*-catenin in the normal mucosa adjacent to the colorectal neoplasms.

Each immunostained section was examined under light microscopy and evaluated by a senior pathologist (TY) according to the modified scoring method of Hugh *et al* (1999) (Figure 2

). Nuclear, cytoplasmic, and membranous stainings were considered separately. Nuclear expression by tumour cells was evaluated as widespread (>75% of cells per section), focal (<75% of cells per section), or absent (no nuclear staining). Cytoplasmic staining was evaluated as negative or positive. Staining intensity of the cell membrane was compared with that seen in normal mucosal cells of colorectum, and then was scored as: 2+ (preserved type), with tumour cells displaying well-localised membranous staining simulating normal mucosa; 1+ (reduced type), with tumour cells displaying reduced membranous immunoreactivity; 0 (absent type), with no immunostaining of tumour cells. Normal colonic epithelial cells were used as an internal control.

### DNA preparation from pure-type SA, CAD, and SCA

To extract genomic DNA, five sections (each 5 *μ*m thick) were obtained from an archival block of formalin-fixed, paraffin-embedded tumour tissue for each polyp type. One section was stained with H&E, and the percentage of tumour cells was estimated by microscopic examination. Representative tumour samples contained a minimum of 80% tumour cells. After deparaffinisation, DNA samples were extracted from the remaining four sections using standard proteinase K–phenol–chloroform methods.

### Laser-capture microdissection for mixed-type SA

To exclude contamination between tumour types, separate samples were obtained from mixed-type SAs for adenomatous areas and other areas (i.e., hyperplastic tissue, conventionally adenomatous tissue, or carcinomatous tissue) using laser-capture microdissection. Deparaffinised sections were stained with H&E, followed by three dehydration steps of 60 s each in 70, 95, and 99.5% ethanol, with final dehydration in xylene. Once air-dried, the stained tissues were laser-capture microdissected by a PixCell II LCM system following the manufacturer's protocols (Acturus Engineering, Mountain View, CA, USA). After the tumour cells had been collected into a 0.5-ml reaction tube, DNA was isolated by adding 50 *μ*l of proteinase K digestion buffer. The reaction tubes were incubated overnight at 37°C. After denaturation at 95°C for 8 min, the lysate was used for DNA analysis.

### Detection of *β*-catenin-gene mutations

Exon 3 of *β*-catenin was examined for mutations using fluorescence-based polymerase chain reaction-single-strand conformation polymorphism (PCR-SSCP) analysis. Primers used for PCR were 5′-TGATTTGATGGAGTTGGACA-3′ (forward) and 5′-CTGTTCCCA-CTCATACAGGA-3′ (reverse). The PCR reaction was carried out in a 20 *μ*l final volume, containing 50 ng DNA, 0.5 U Takara Ex Taq (Takara, Sigma, Japan), 1 *μ*mol of each primer, 2.5 mM of each dNTP, and 10 × Ex Taq buffer (Takara). The following cycling protocol was used: 94°C for 1 min, followed by 40 cycles of 94°C for 30 s, 55°C for 1 min, and 72°C for 1 min, and then a final extension at 10°C for 60 min. PCR products were visualised and purified by electrophoresis on 2% agarose gels for 40 min at 100 V, and stained with ethidium bromide. These PCR products were used to detect *β*-catenin mutation by fluorescence-based SSCP analysis using an automated DNA sequencer (ALF Express; Amersham Pharmacia Biotech, Uppsala, Sweden), with an external cooling bath. Peak patterns were analysed using the ALFwin Fragment Analyser program (Amersham Pharmacia Biotech). Shifted peaks indicated the mutations in the DNA fragments. Nucleotide sequences of DNA fragments with shifted peaks were determined as described previously (Makino *et al*, 2000).

### Statistical analysis

To determine statistical significance, relation between molecular and clinicopathologic data were analysed using *χ*^2^ test, or Fisher's exact test as appropriate. A value of *P* less than 0.05 was considered to indicate significance.

## RESULTS

### Expression of *β*-catenin in normal colonic mucosa

Membranous staining of *β*-catenin uniformly localised at the intercellular borders was observed in the normal mucosa adjacent to colorectal neoplasms (Figure 2B). No nuclear or cytoplasmic immunostaining was seen in normal mucosa.

### Nuclear expression of *β*-catenin

Table 2

**Table 2:** Nuclear expression of *β*-catenin in SAs, CADs, and SCAs

**Histology**	**Absent**	**Focal**	**Widespread**	**Total**
SAs*				
<10 mm	18 (100%)	0	0	18
⩾10 mm	24 (89%)	3 (11%)	0	27
				
CADs*				
<10 mm	18 (46%)	19 (49%)	2 (5%)	39
⩾10 mm	10 (31%)	19 (59%)	3 (9%)	32
				
SCAs	1 (12%)	3 (38%)	4 (50%)	8

* *P*<0.0001. SAs=serrated adenomas; CADs=conventional adenomas; SCAs=carcinomas invading the submucosa.

shows nuclear expression of *β*-catenin in SAs, CADs, and SCAs. No widespread immunostaining was observed in the nuclei of SA cells, with nuclear expression being restricted to focal areas. Widespread or focal nuclear expression of *β*-catenin was demonstrated in only 7% of SAs (three out of 45), in contrast to 61% of CADs (43 out of 71) (*P*<0.0001). All but one SCA specimen was positive for nuclear staining. In SAs and CADs, nuclear expression of *β*-catenin was observed more frequently in large (>10 mm) than in small (<10 mm) lesions. However, these size-related differences were not statistically significant.

### Cytoplasmic and cell-membranous expression of *β*-catenin

Table 3

**Table 3:** Cytoplasmic expression of *β*-catenin in SAs, CADs, and SCAs

**Histology**	**Negative**	**Positive**	**Total**
SAs*			
<10 mm	16 (89%)	2 (11%)	18
⩾10 mm	22 (81%)	5 (19%)	27
			
CADs*			
<10 mm**	14 (36%)	25 (64%)	39
⩾10 mm**	2 (6%)	30 (94%)	32
			
SCAs	1 (12%)	7 (88%)	8

* *P*<0.0001;

** *P*=0.0029. SAs=serrated adenomas; CADs=conventional adenomas; SCAs=carcinomas invading the submucosa.

shows cytoplasmic expression of *β*-catenin in SAs, CADs, and SCAs. Cytoplasmic accumulation of *β*-catenin was demonstrated in 16% of SAs (seven out of 45) and 77% of CADs (55 out of 71: *P*<0.0001). All but one SCA specimen was positive for cytoplasmic staining. In CADs, cytoplasmic immunostaining for *β*-catenin was detected more frequently in large (⩾10 mm) than in small (<10 mm) CADs (*P*=0.0029). However, no significant difference was noted in cytoplasmic immunostaining of *β*-catenin between large and small SAs. No significant difference in cell-membrane expression of *β*-catenin could be found among SAs, CADs, and SCAs.

### Detection of *β*-catenin mutation

No mutation in exon 3 of *β*-catenin was found in SAs or SCAs, while 7% of CADs (five out of 71) had *β*-catenin mutations. Considering the size of CADs, *β*-catenin mutations were detected in 8% of small CADs (<10 mm; three out of 39) and 6% of large CADs (>10 mm; two out of 32). Table 4

**Table 4:** Characteristics of colorectal neoplasms with *β*-catenin mutation

**Sample no.**	**Location**	**Size (mm)**	**Sequence change**	**Codon**	**Amino-acid substitution**
Small CAD (<10 mm)					
AD59	T	5	TCT to TTT	45	Ser to Phe
AD60	T	5	TCT to TTT	45	Ser to Phe
AD71	T	3	TCT to TTT	45	Ser to Phe
					
Large CAD (⩾10 mm)					
6207	C	18	TCT to CCT	37	Ser to Pro
4799	Rs	40	TCT to CCT	37	Ser to Pro

CAD=Conventional adenoma; T=transverse colon; C=caecum; Rs=rectosigmoid.

summarises tumour location, tumour size, substitution of one base, codon affected, and predicted amino-acid substitution. All mutations involved loss of serine or threonine residues from the GSK-3*β* phosphorylation region.

### Immunohistochemical and genetic findings of *β*-catenin in mixed-type SAs

**Figure 3:**
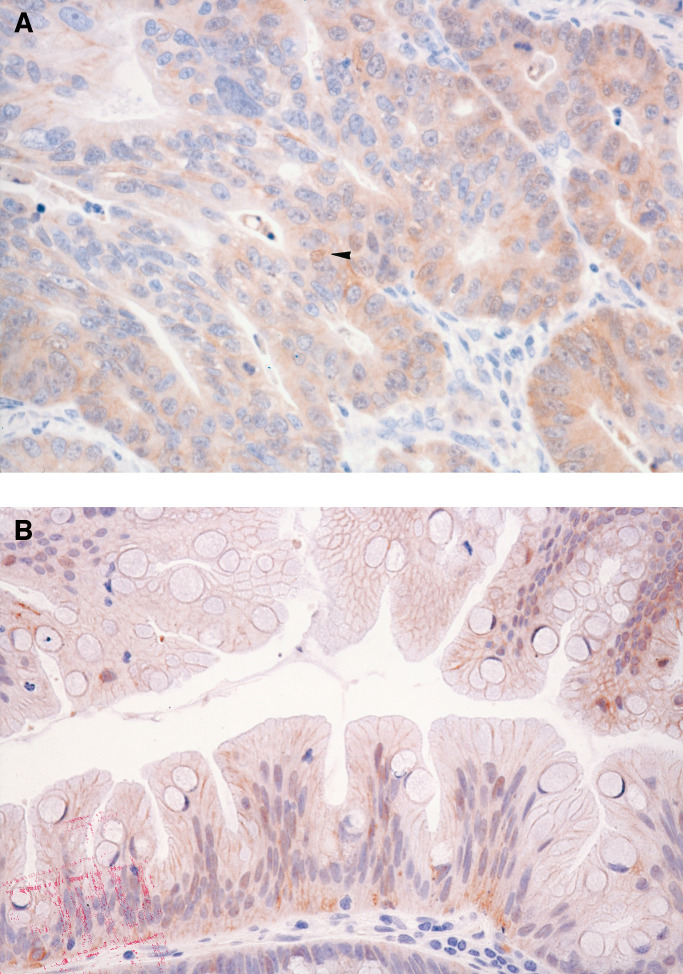
Immunohistochemical findings in a Dukes' A carcinoma associated with SA. The carcinoma invaded the submucosal layer. In (**A**), focal nuclear staining of cells for *β*-catenin is detected; and cytoplasmic staining for *β*-catenin is observed in the carcinomatous epithelium of this mixed-type SA. In (**B**), neither nuclear nor cytoplasmic staining for *β*-catenin is found in the adjacent serrated epithelium.

In the 21 mixed-type SAs, serrated and nonserrated areas were identified histologically as shown in Table 1. The 21 mixed-type SAs involved various nonserrated components as follows: 13 hyperplastic polyps, seven CAs, and one carcinoma that invaded the submucosal layer. No nuclear or cytoplasmic immunostaining for *β*-catenin was observed in the hyperplastic or conventionally adenomatous epithelium in the 20 mixed polyps. In the carcinomatous epithelium of mixed-type SAs, nuclear *β*-catenin-positive cells were focally detected, and cytoplasmic staining for *β*-catenin was observed (Figure 3

). Neither nuclear nor cytoplasmic staining for *β*-catenin was found in the adjacent serrated epithelium. Microdissection allowed each area of mixed-type SA to be examined separately for a *β*-catenin mutation. However, no *β*-catenin mutation was found in serrated or nonserrated areas.

## DISCUSSION

Recent reports have described nuclear expression of *β*-catenin in familial and sporadic colorectal tumours (Inomata *et al*, 1996; Takayama *et al*, 1996; Hao *et al*, 1997; Valizadeh *et al*, 1997; Hugh *et al*, 1999; Kobayashi *et al*, 2000). Valizadeh *et al* (1997) have suggested that both cytoplasmic and nuclear localisation of *β*-catenin in dysplastic colonic polyps are early events in the development of colorectal cancer. Nuclear localisation has been linked to progression of colorectal carcinogenesis when a certain threshold tumour size is exceeded (Brabletz *et al*, 2000). However, Kobayashi *et al* (2000) have reported that nuclear translocation of *β*-catenin is involved in initiation of cancer in distinction to adenoma, independently of *APC* mutations. In our study, about 80% of CADs displayed either cytoplasmic or nuclear localisation of *β*-catenin, while nuclear overexpression was observed in 50% of SCAs (four out of eight). Abnormal distribution of *β*-catenin was detected more frequently in large CADs (⩾10 mm) than in smaller CADs. However, nuclear overexpression of *β*-catenin generally was not observed in our SAs; even focal nuclear staining was found in only 7% (three out of 45). Cytoplasmic accumulation of *β*-catenin was observed in 16% of SAs (seven out of 45). The nuclear and cytoplasmic localisation of *β*-catenin in SA thus differs from observations in CADs and SCAs.

We identified mutations in exon 3 of *β*-catenin in 7% of sporadic CADs (five out of 71). As in previous studies (Kitaeva *et al*, 1997; Morin *et al*, 1997; Sparks *et al*, 1998; Samowitz *et al*, 1999), all mutations involved the loss of serine or threonine residues from GSK-3*β* phosphorylation sites. Loss of these phosphorylation sites is thought to promote tumorigenesis through decreased APC-associated degradation of *β*-catenin and increased *β*-catenin/Tcf transcriptional activation (Morin *et al*, 1997).

One important reported finding in CADs is a difference in the incidence of *β*-catenin mutation between small and large CADs. Samowitz *et al* (1999) have suggested that adenomas with mutations in exon 3 of *β*-catenin are unlikely to progress to large adenomas or invasive cancers, since *β*-catenin mutations are significantly more common in very small adenomas than in large adenomas or invasive cancers. Mutations in *β*-catenin sometimes were seen in our large CADs as well as small CADs, with no significant difference in frequency according to lesion size (small, 6%; large, 8%). This disagreement with the previous study (Samowitz *et al*, 1999) suggests that mutation of *β*-catenin may be an early, nonsize-dependent event in the conventional adenoma–carcinoma sequence.

*β*-Catenin mutation occurs in primary CRC and CRC cell lines that lack APC mutation (Morin *et al*, 1997). *APC* mutation is infrequent in MSI-H cancers (Konishi *et al*, 1996; Salahshor *et al*, 1999). However, *β*-catenin mutation is not found in sporadic CRC with high level of microsatellite instability (MSI-H) (Salahshor *et al*, 1999). These observations fit with the lack of abnormal *β*-catenin immunostaining in sporadic MSI-H cancers (Wong *et al*, 2002). When *β*-catenin mutation is found in sporadic MSI-H cancers, the subjects are young and probably have HNPCC (Mirabelli-Primdahl *et al*, 1999). Moreover, Mirabelli-Primdahl *et al* (1999) have reported that *β*-catenin mutation is not found in 27 samples of microsatellite stable CRC. It can therefore be inferred that *β*-catenin mutation is implicated in only a very small proportion of CRC that may be a subset of HNPCC cases.

Recent experimental and clinical analyses have indicated that cytoplasmic expression and nuclear translocation of *β*-catenin in colorectal tumour cells usually results from mutations in the *APC* or *β*-catenin genes (Morin *et al*, 1997; Rubinfeld *et al*, 1997; Iwao *et al*, 1998). Unregulated intracellular *β*-catenin protein has been shown to activate its target gene expression by the complex formation with a family of nuclear transcription factors. *APC* mutations are present in about 80% of sporadic colorectal adenomas and cancers, while in recent studies (Uchida *et al*, 1998; Dehari, 2001), *APC* mutations have been rare in SAs. We observed no mutation of *β*-catenin in SAs. Nuclear or cytoplasmic expression of *β*-catenin was significantly less common in SAs than in CADs or SCAs. Furthermore, no nuclear or cytoplasmic immunostaining of *β*-catenin was observed in adjacent hyperplastic or conventionally adenomatous glands in mixed-type SAs. Thus, the signalling pathway involving APC and *β*-catenin seems unlikely to be important for tumorigenesis in most SAs.

The previous study (Samowitz *et al*, 1999) has suggested that *β*-catenin mutation occurs in a small subset of CAD, and CAD initiated by *β*-catenin mutation may rarely progress to CRC. From the preceding observation, it can be deduced that *β*-catenin is not implicated in the initiation of SAs, or that SAs rarely progress to carcinoma. We demonstrated that no mutation of *β*-catenin was detected in SAs. Furthermore, when we separately examined *β*-catenin mutation in serrated and nonserrated areas of mixed-type SAs, no *β*-catenin mutation was found in either area. Thus, mutations in exon 3 of *β*-catenin are unlikely to contribute to tumorigenesis in SA. In fact, it has been previously shown that *β*-catenin and *APC* mutations are uncommon in SAs (Sawyer *et al*, 2002). This supports the suggested role of SAs in the pathogenesis of sporadic MSI-H cancer.

We found one mixed-type SA with two histologically distinct areas representing carcinoma and SA. Although focally nuclear expression of *β*-catenin was demonstrated in the carcinomatous component of this lesion, cytoplasmic accumulation of *β*-catenin was observed. Neither nuclear nor cytoplasmic staining was found in the adjacent serrated epithelium. The immunohistologic heterogeneity demonstrated in this tumour implies that intracellular redistribution of *β*-catenin might be a late event in the development of colorectal cancer. However, only one such case of carcinoma associated with SA was analysed in this study, so further investigation of these genetic changes is required to clarify the mechanism of tumorigenesis in SA.

In summary, we evaluated the status of *β*-catenin in the development of SA using immunohistochemical and genetic methods. Intracellular localisation of *β*-catenin in SAs differed from that in CADs or SCAs, and no *β*-catenin mutation was observed in SAs. We concluded that *β*-catenin mutation is unlikely to contribute to tumorigenesis in SA. Further, intracellular localisation of *β*-catenin may not be associated with an early tumorigenic event in most SAs.
